# Budd-Chiari Syndrome: A Case Report of a Rare Presentation of COVID-19

**DOI:** 10.7759/cureus.12554

**Published:** 2021-01-07

**Authors:** Azhar A Sh. Hassan, Mujtaba E Alsaleh, Muntadher E Alsaleh, Fatimah A Al Zaher, Fatema A Almajed, Ahmed M Alkhudhair, Maram M Alali, Hassan A Alzayer, Areej J Alolayan

**Affiliations:** 1 Family and Community Medicine, Imam Abdulrahman Bin Faisal University, Dammam, SAU; 2 Internal Medicine, King Faisal University, Hofuf, SAU; 3 Internal Medicine, King Fahad Hospital in Alhofuf, Hofuf, SAU; 4 Internal Medicine, Jazan University, Jazan, SAU; 5 Internal Medicine, Imam Abdulrahman Bin Faisal University, Dammam, SAU; 6 Internal Medicine, Medical University of Warsaw, Warsaw, POL

**Keywords:** covid-19, covid coagulopathy, budd-chiari syndrome

## Abstract

Coronavirus Disease 2019 (COVID-19) predominantly involves the respiratory system and shows a wide range of severity. There is a growing body of evidence about the occurrence of thromboembolic events in COVID-19. Case Report: We report the case of a 48-year-old female patient who presented with sudden-onset abdominal pain. Physical examination revealed ascites and tender hepatomegaly. Subsequently, abdominal computed tomography was performed which revealed thrombosis in the hepatic vein and inferior vena cava in keeping with Budd-Chiari Syndrome. The patient was started on low-molecular-weight heparin and supportive care. Clinical improvement was observed over the course of the treatment and the patient was discharged after 10 days from the presentation. Thromboembolic events could be the first manifestation of COVID-19. Early recognition of these complications is crucial for prompt management.

## Introduction

Coronavirus Disease 2019 (COVID-19) primarily involves the respiratory system and shows a wide spectrum of severity ranging from asymptomatic to acute respiratory distress syndrome. However, extra-pulmonary manifestations of COVID-19 are gaining attention. It is reported that up to one-third of patients with COVID-19 exhibit coagulopathy [1]. The spectrum of coagulopathy involves venous and arterial events [1, 2].

Budd-Chiari syndrome develops due to an obstruction in the hepatic venous outflow. It has an incidence rate of 2.0 per million population. We present the case of a middle-aged woman with COVID-19 who presented in a clinical picture of Budd-Chiari Syndrome [3]. To the best of our knowledge, this is the first report of a patient with COVID-19 who developed this complication.

## Case presentation

We report the case of a 48-year-old female patient who presented to the emergency department complaining of diffuse abdominal pain which had been ongoing for three days and which was associated with progressive abdominal distension. The pain started suddenly and was increasing in severity. She rated the severity as 8 out of 10 on presentation. The pain did not radiate and has no exacerbating or relieving factors. She also reported having a decreased appetite. She did not report a history of nausea/vomiting, fever, or change in bowel or urinary habits. All other systems reviewed were non-contributory. The past medical history is remarkable for long-standing hypertension that is well-controlled with amlodipine 5 mg once a day. The patient was not on oral contraceptive pills or hormonal therapy. She had not undergone any abdominal surgeries. She did not smoke cigarettes and had no history of alcohol drinking.

On examination, the patient looked in distress. She was not pale or icteric. Her vital signs were as follows: pulse rate of 120 bpm, blood pressure of 130/84 mmHg, respiratory rate of 14 bpm, and a temperature of 37.1 °C. Abdominal examination revealed a generalized tenderness without clinical signs of peritonitis. Mild ascites and enlarged tender liver were noted. Chest examination revealed scattered crackles.

Basic hematological investigation revealed a hemoglobin of 13.5 g/dL, a leucocyte of 8 × 103/mL, and a platelet count of 350,000/mL. As per our institution policy, the patient underwent a polymerase chain reaction (PCR)-based nasopharyngeal swab testing for COVID-19 which revealed a positive result. There was a marked elevation in the liver enzymes, including alanine transferase (1325 U/L), aspartate transferase (1344 U/L), alkaline phosphatase (605 U/L), total bilirubin (2.1 mg/dL), and international normalized ratio (1.6). Subsequently, the viral hepatitis serology panel yielded negative results.

**Figure 1:**
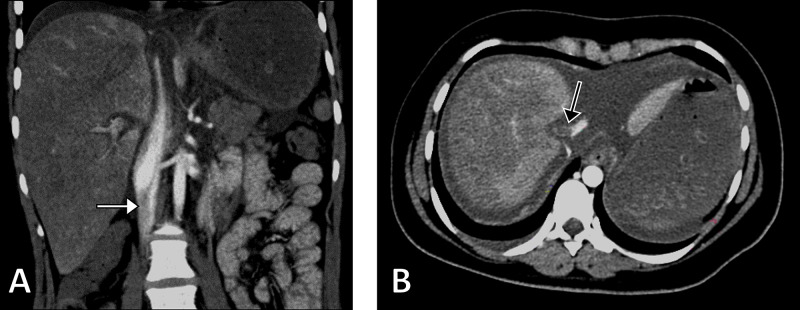
Computed Tomography Images Coronal (A) and axial (B) computed tomography images demonstrating a filling defect in the inferior vena cava (white arrow) and the hepatic vein (black arrow).

In light of the clinical and radiological findings, the patient underwent a chest X-ray which revealed a bilateral patchy opacification in keeping with mild COVID-19 pneumonia. Furthermore, contrast-enhanced abdominal computed tomography revealed filling defects in the inferior vena cava and hepatic vein and enlarged non-homogenous liver (Figure 1).

Anticoagulant therapy with low molecular weight heparin (1 mg/kg/day) was immediately initiated along with supportive treatment, including diuretics. A comprehensive thrombophilia screen was performed and the results were negative. The patient demonstrated improvement in the clinical and laboratory parameters. She was discharged on oral warfarin after a hospital stay of 10 days. Results of repeated follow-up PCR tests for COVID-19 were negative. She was asymptomatic in the follow-up visit after one month from discharge.

## Discussion

To our knowledge, this is the first case of a patient of COVID-19 presenting with Budd-Chiari Syndrome. There is a growing body of evidence about coagulopathy in COVID-19. While the pathogenesis remains not fully understood, hypercoagulation can be viewed in the context of Virchow’s triad, which includes endothelial injury, stasis, and hypercoagulable state.

Several studies revealed changes in the level of coagulation factors in patients with COVID-19, such as elevated factor VIII and fibrinogen levels [4, 5]. Autopsy studies in patients who died from COVID-19 revealed significant generalized thrombotic microangiopathy [6].

The spectrum of coagulopathy in COVID-19 is wide and includes venous thromboembolism, arterial events, and microvascular thrombosis [7-9]. Evidence suggests that hemostasis dysregulation is more evident in patients with severe COVID-19 [7]. However, the present case demonstrated an example that coagulopathy may occur even in patients with mild COVID-19.

The guidelines for the management of coagulopathy in COVID-19 are not clear. In spite of patients being on optimal physician-directed anti-coagulant therapy, thrombotic events are known to occur [10].

## Conclusions

Budd-Chiari syndrome should be borne in mind as a possible complication of COVID-19. Thromboembolic events could be the first manifestation of COVID-19. Early recognition of these complications is crucial for prompt management and better prognosis.
